# Supplementary material for: Imatinib disassembles the regulatory core of Abelson kinase by binding to its ATP site and not by binding to its myristoyl pocket
Source: Magn Reson (Gott). 2022 May 20;3(1):91–9. doi: 10.5194/mr-3-91-2022 (PMC10539847; doi:10.5194/mr-3-91-2022)
Supplement: The supplement related to this article is available online at: https://doi.org/10.5194/mr-3-91-2022-supplement. [file mr-3-91-supplement.pdf]

Supplement of Magn. Reson., 3, 91–99, 2022
https://doi.org/10.5194/mr-3-91-2022-supplement
© Author(s) 2022. CC BY 4.0 License.

Supplement of

Imatinib disassembles the regulatory core of Abelson kinase by binding to its ATP site and not by binding to its myristoyl pocket

Stephan Grzesiek et al.

Correspondence to: Stephan Grzesiek (stephan.grzesiek@unibas.ch)

The copyright of individual parts of the supplement might differ from the article licence.

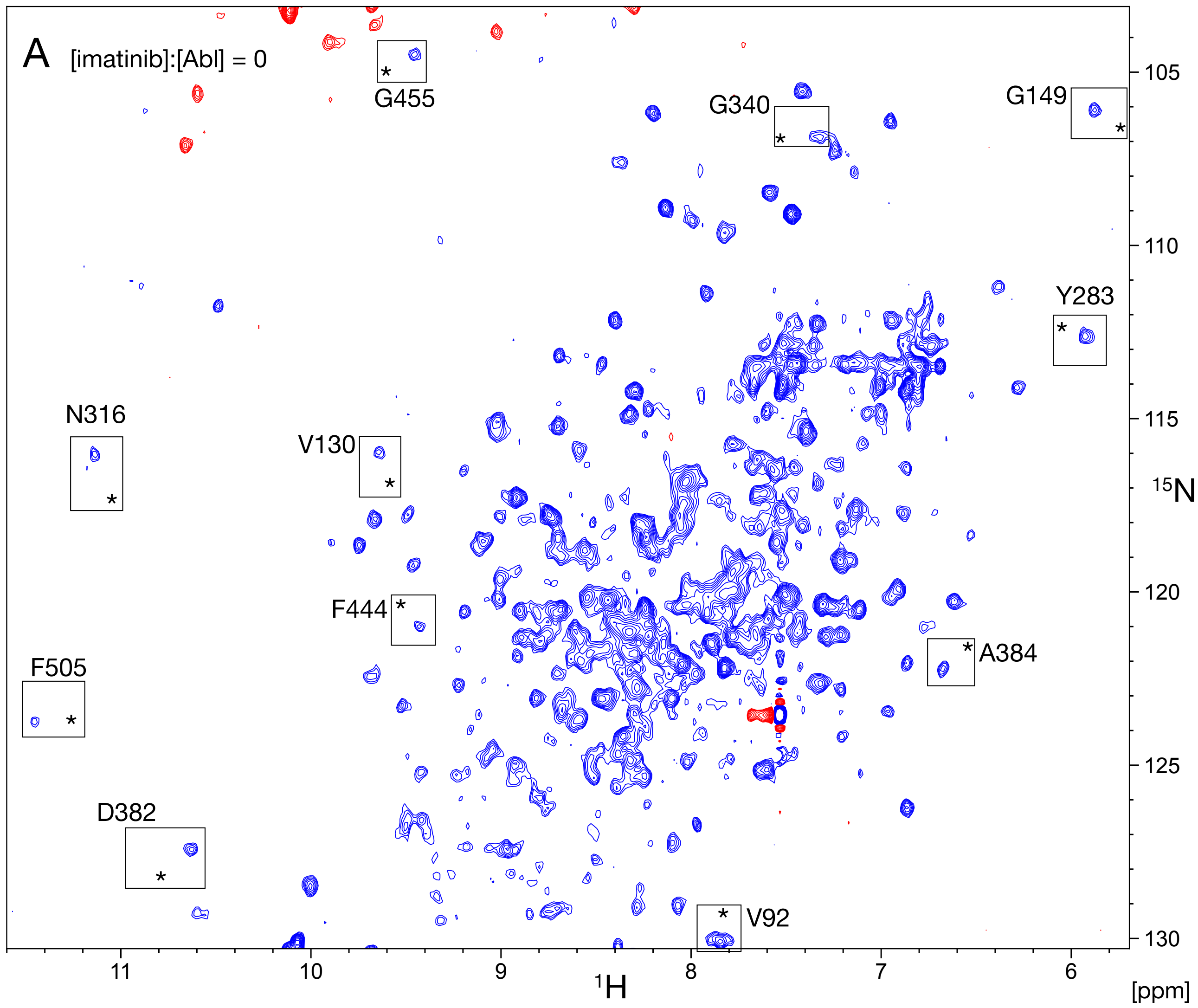

Figure S1 continued overleaf

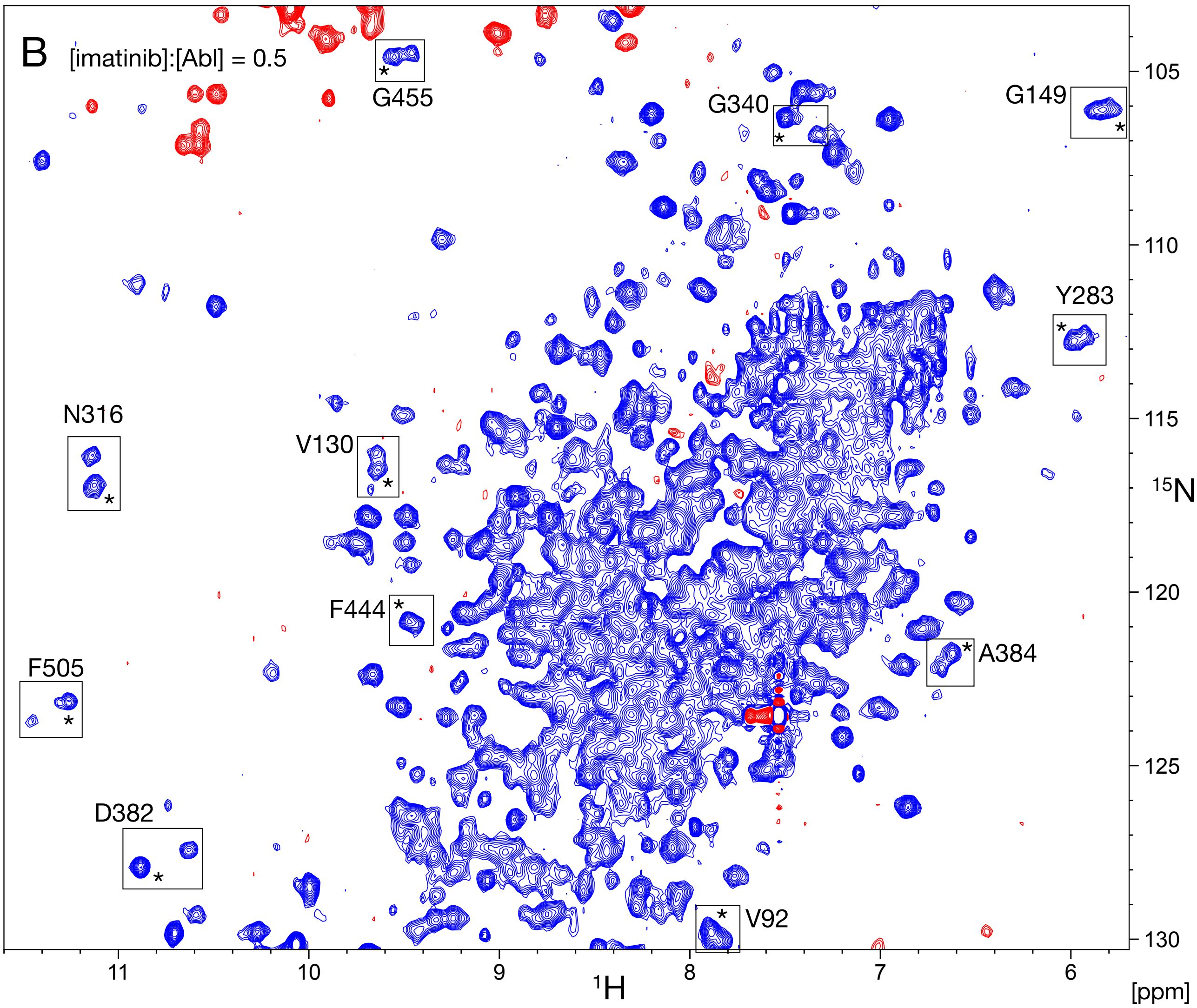

Figure S1 continued overleaf

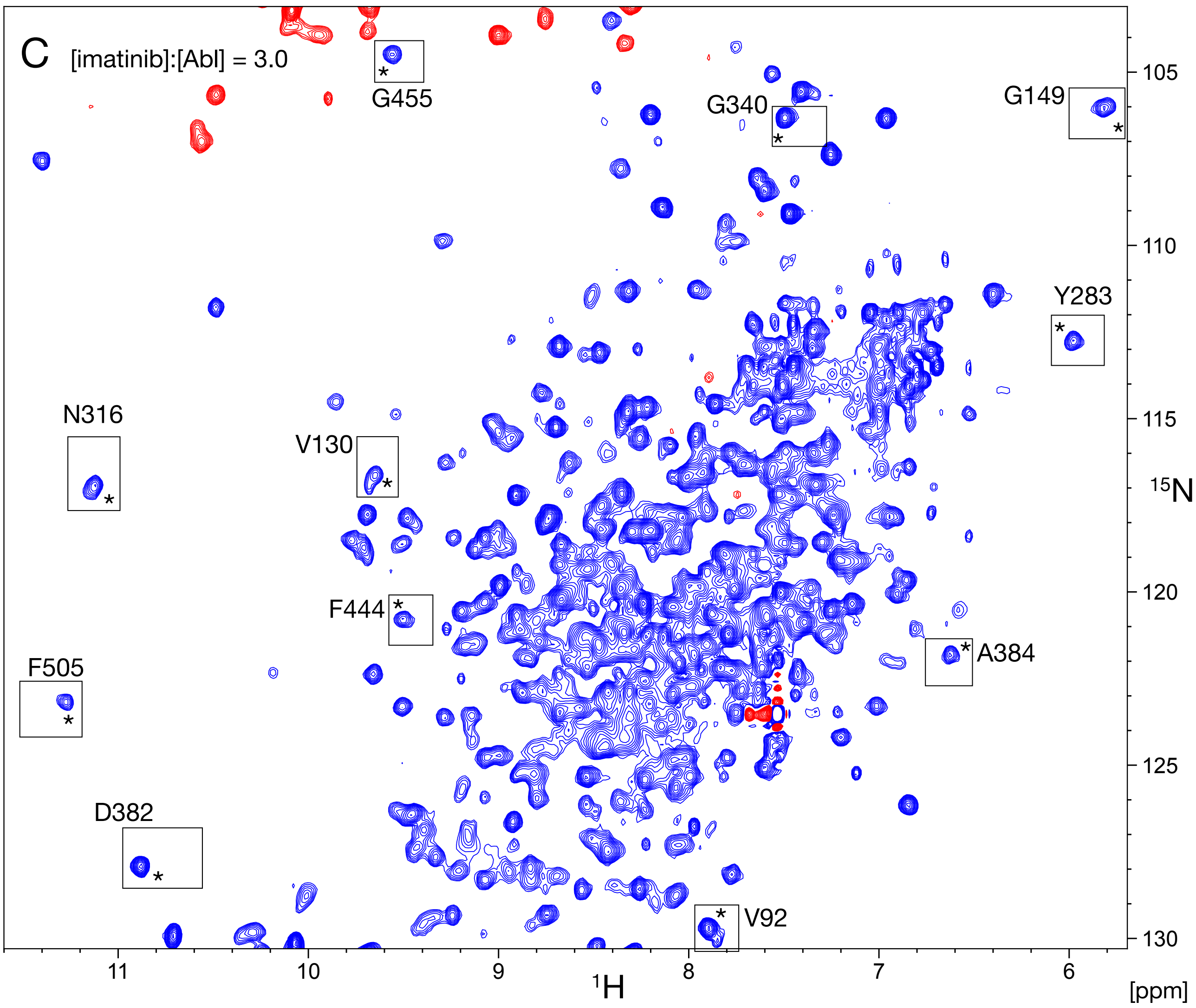

Figure S1. Full ^{1}H-^{15}N TROSY spectra of the Abl$^{83\text{-}534}$ core at various molar imatinib:Abl$^{83\text{-}534}$ ratios. The panels depict the full spectral range, from which the regions in Figure 2A were selected. [imatinib]:[Abl$^{83\text{-}534}$] = 0 (panel A), 0.5 (panel B), 3.0 (panel C). Selected resonances from residues in the SH3 (V92, V130), SH2 (G149), and kinase domain (Y283, N316, G340, D382, A384, F444, G455, F505), which clearly differ in their apo and imatinib-bound states, are marked by rectangles and assignment information. The resonances of the imatinib-bound state are marked by an asterisk.
